# Some of the most interesting CASP11 targets through the eyes of their authors

**DOI:** 10.1002/prot.24942

**Published:** 2015-11-16

**Authors:** Andriy Kryshtafovych, John Moult, Arnaud Baslé, Alex Burgin, Timothy K. Craig, Robert A. Edwards, Deborah Fass, Marcus D. Hartmann, Mateusz Korycinski, Richard J. Lewis, Donald Lorimer, Andrei N. Lupas, Janet Newman, Thomas S. Peat, Kurt H. Piepenbrink, Janani Prahlad, Mark J. van Raaij, Forest Rohwer, Anca M. Segall, Victor Seguritan, Eric J. Sundberg, Abhimanyu K. Singh, Mark A. Wilson, Torsten Schwede

**Affiliations:** ^1^ Genome Center, University of CaliforniaDavisCalifornia95616; ^2^ Department of Cell Biology and Molecular GeneticsInstitute for Bioscience and Biotechnology Research, University of MarylandRockvilleMaryland20850; ^3^ Institute for Cell and Molecular Biosciences, University of NewcastleNewcastle upon TyneNE2 4HHUnited Kingdom; ^4^ Broad InstituteCambridgeMassachusetts02142; ^5^ TimPharmaSanta ClaritaCalifornia91350; ^6^ Department of BiologySan Diego State UniversitySan DiegoCalifornia92182; ^7^ Department of Computer ScienceSan Diego State UniversitySan DiegoCalifornia92182; ^8^ Department of Structural BiologyWeizmann Institute of ScienceRehovot76100Israel; ^9^ Department of Protein EvolutionMax Planck Institute for Developmental BiologyTübingen72076Germany; ^10^ BerylliumBainbridge IslandWashington D.C98110; ^11^ Biomedical Manufacturing Program, CSIROParkvilleVICAustralia; ^12^ Institute of Human Virology, University of Maryland School of MedicineBaltimoreMaryland21201; ^13^ Department of Biochemistry and Redox Biology CenterUniversity of Nebraska‐LincolnLincolnNebraska68588; ^14^ Centro Nactional De Biotecnologia (CNB‐CSIC)MadridE‐28049Spain; ^15^ Department of Biology and Viral Information InstituteSan Diego State UniversitySan DiegoCalifornia92182; ^16^ Human Longevity IncLa JollaCalifornia92121; ^17^ Institute of Human Virology, University of Maryland School of MedicineBaltimoreMaryland21201; ^18^ Department of MedicineUniversity of Maryland School of MedicineBaltimoreMaryland21201; ^19^ Department of Microbiology and ImmunologyUniversity of Maryland School of MedicineBaltimoreMaryland21201; ^20^ School of BiosciencesUniversity of KentCanterburyKentUnited Kingdom; ^21^ Biozentrum, University of BaselBasel4056Switzerland; ^22^ SIB Swiss Institute of BioinformaticsBasel4056Switzerland

**Keywords:** X‐ray crystallography, NMR, CASP, protein structure prediction

## Abstract

The Critical Assessment of protein Structure Prediction (CASP) experiment would not have been possible without the prediction targets provided by the experimental structural biology community. In this article, selected crystallographers providing targets for the CASP11 experiment discuss the functional and biological significance of the target proteins, highlight their most interesting structural features, and assess whether these features were correctly reproduced in the predictions submitted to CASP11. Proteins 2016; 84(Suppl 1):34–50. © 2015 The Authors. Proteins: Structure, Function, and Bioinformatics Published by Wiley Periodicals, Inc.

**Abbreviations**
CASP: community wide experiment on the critical assessment of techniques for protein structure prediction
SLC: solute carrier family
STAC: SLC5 and TCST‐associated component
TCST: Two‐component signal transduction system.

## INTRODUCTION

The community‐wide experiment on the Critical Assessment of Techniques for Protein Structure Prediction (CASP) provides an independent mechanism for assessing methods in protein structure prediction.^1^ The experiment has a reputation of an unbiased testing ground, with the credibility of results ensured through the “blind prediction” principle requesting all predictions to be made on proteins with hitherto unknown structures. To get a supply of modeling targets, the CASP organization relies on the help of the experimental structural biology community. Since CASP started in 1994, the community has provided >850 sequences of soon‐to‐be‐solved protein structures as prediction targets, including 100 sequences offered for the latest, 11th round of CASP. Of these, 56 targets were from the Structural Genomic centers, and the remaining 44 from non‐SGI research centers and other research groups. In addition to these, 27 targets have been submitted to CASP Roll in between the biennial CASP10 and CASP11 experiments.

This manuscript is the third in a series of articles^2^, ^3^ where experimentalists describe the most interesting aspects of the targets provided to CASP and assess to what extent these aspects were correctly reproduced in the predictions. The chapters of the article reflect the views of the contributing authors and discuss the following proteins: YaaA—the first characterized member of the DUF328 family of proteins, which was extraordinary well predicted in CASP11; the L4 domain of the laminin protein; the snake adenovirus 1 fiber head; a novel biofilm‐dispersing nuclease; a new protein domain associated with transmembrane solute transport and two component signal transduction; a monotreme lactation protein MLP and a human vanin protein; an unknown phage protein from the marine environment; and the major Type IV pilin of *Clostridium difficile* NAP08.

The results of the comprehensive numerical evaluation^4^ of all CASP11 models are available at the Prediction Center website (http://www.predictioncenter.org); the detailed assessment of the models by the human assessors is provided in dedicated manuscripts elsewhere in this issue.

### *Escherichia coli* YaaA, the first characterized member of the DUF328 proteins (CASP: T0806; PDB: 5CAJ)—provided by Janani Prahlad and Mark A. Wilson

Molecular oxygen is both essential for metabolism in aerobic organisms and easily converted into reactive oxygen species (ROS) that can damage the cell. The excessive production of ROS causes oxidative stress, which all organisms (even anaerobes that only rarely contact oxygen) must combat. A great deal is known about how cells defend themselves against oxidative stress, with prokaryotes being especially well‐studied. Nevertheless, some prokaryotic proteins that are part of the oxidative stress response are still functionally uncharacterized. One such protein is the *Escherichia coli* protein YaaA (gene b0006).

YaaA is a 30 kDa member of the DUF328/UPF0246 family of proteins. Abundant in bacteria but rare in archaea and eukaryotes, the molecular function of these proteins is unknown. In contrast, the cellular function of the DUF328 proteins has been initially characterized in a recent study of the *E. coli* member YaaA.^5^ The transcription of YaaA is regulated by the OxyR peroxide‐responsive transcription factor, identifying YaaA as a component of the bacterial oxidative stress response. Although deficiency of YaaA does not produce a phenotype in laboratory *E. coli* strain MG1655 under normal growth conditions, a severe growth defect is apparent in *E. coli* that have been engineered to accumulate micromolar levels of hydrogen peroxide under basal growth conditions (Hpx‐ *E. coli*). The poor growth phenotype of YaaA‐deficient *E. coli* is most evident when Hpx‐ cells are grown anaerobically (*E. coli* is a facultative anaerobe) and then moved into aerobic atmosphere, where they stop dividing and adopt a highly filamentous morphology indicative of extreme stress.

The basis of this growth deficit appears to be that YaaA‐ *E. coli* accumulate higher levels of intracellular Fe^2+^, which is a dangerous cation in combination with hydrogen peroxide due to the production of the highly reactive hydroxyl radical (.OH) through Fenton chemistry. In addition, the absence of YaaA leads to a higher rate of mutations than observed in wild‐type cells, indicating a potential role for YaaA in DNA protection or repair. This DNA‐related hypothesis is further supported by the growth defects of YaaA‐ *E. coli* that have nonfunctional RecA: this phenotype is apparent even in cells that can effectively scavenge ROS. Considered in total, YaaA appears to play an important role in managing bacterial oxidative stress and is connected to both intracellular iron levels and DNA integrity^5^.

**Figure 1:**
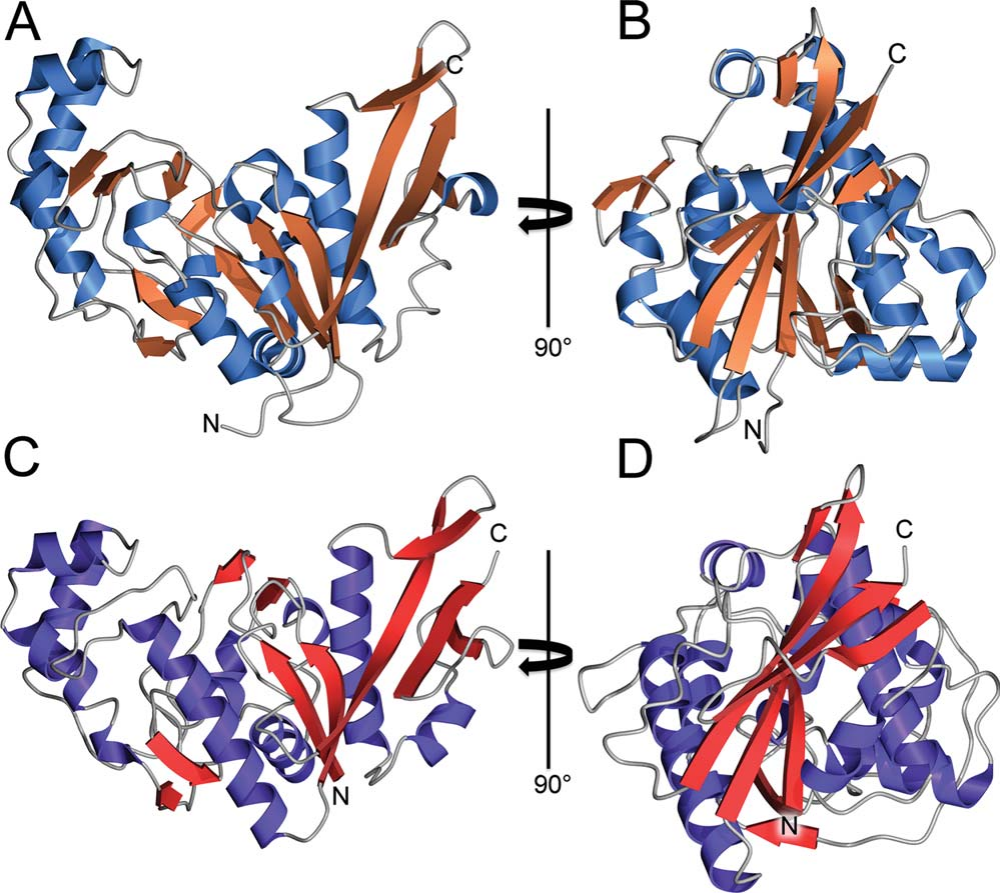
Experimental and predicted structures of *E. coli* YaaA. (**A** and **B**) The experimentally determined crystal structure shown as a ribbon diagram, with β‐strands colored orange and α‐helices blue. YaaA possesses a new fold and has an apical depression that is rich in basic residues. (**C**, **D**) CASP model T0806TS064_1‐D1 is shown in the same orientation as the experimental structure in panels A and B. The excellent overall agreement between experiment and prediction is apparent. In some areas, relatively minor differences in backbone torsion angles result in differing secondary structure assignments.

The structure of YaaA has been determined to 1.65 Å resolution using X‐ray crystallography. As expected based on the absence of homology with known structures, YaaA possesses a new fold. The molecule is monomeric and has an overall shape reminiscent of a slice of melon, featuring an apical depression atop a wedge‐shaped protein (Fig. 1). The electrostatic potential in the apical depression is strongly positive due to a number of well‐conserved basic residues that are clustered in this region, suggestive of an anion binding site. A further potential clue about its molecular function is that the protein co‐purifies with large amounts of double stranded DNA that cannot be easily separated by standard protocols for nucleic acid removal such as anion exchange chromatography. Although YaaA retains this DNA during purification, the crystallized protein does not have any electron density consistent with nucleic acid, and dissolved crystals lack nucleic acid.

YaaA is the first structurally characterized member of the DUF328/UPF0246 family and presents an especially challenging target for structure prediction as there are no homologs that can serve as templates. Nevertheless, David Baker's group produced an excellent model (T0806TS064_1‐D1) that correctly predicted the key features of the YaaA fold, including all of the core secondary structural elements with correct topology. No other CASP participant produced a model of comparable quality. We believe that this success can be attributed to the specifics of the underlying prediction method, which effectively used information from the evolutionary constraints.^6^, ^7^ The area in which the predicted model diverges most from the experimental structure is residues 108 to 122, which are two antiparallel β‐strands in the crystal structure but were predicted to be largely α‐helical in the model. The GDT_TS score between the experimentally determined and predicted structure is 60.7, corresponding to a Cα RMSD of 3.6 Å. This agreement is remarkably good given the novel fold and unusually large amount of non‐standard secondary structure in YaaA. The large stretches of nonstandard secondary structure are some of the most unusual aspects of YaaA, and thus it is noteworthy that the Baker group successfully identified these regions (7–28, 68–85, 122–136) as being neither helix nor strand. The regions of non‐standard secondary structure in the predicted model have an average Cα RMSD of 2.9 Å with the crystal structure, which is also quite impressive as there are presumably few template structures available for these atypical regions. After CASP, we tried to phase the X‐ray diffraction data for this target by molecular replacement using the Baker group model. Although we did not succeed, the model's 3.0 Å Cα RMSD with the experimental structure suggests that electron density‐guided structure optimization^8^ may have been feasible in this case. The ability to predict suitable molecular replacement search models for most crystallized proteins would be a major triumph in protein structure prediction and would facilitate experimental structure determination. Furthermore, the successful prediction of the novel YaaA fold highlights the rapid pace of advances being made in structure prediction and gives hope that it may be possible to predict new folds from genomic data alone in the near future.

### Sugar‐binding fold domains decorate the arms of the laminin heterotrimer (CASP: T0812; PDB: 4YEP, 4YEQ)—provided by Deborah Fass

**Figure 2:**
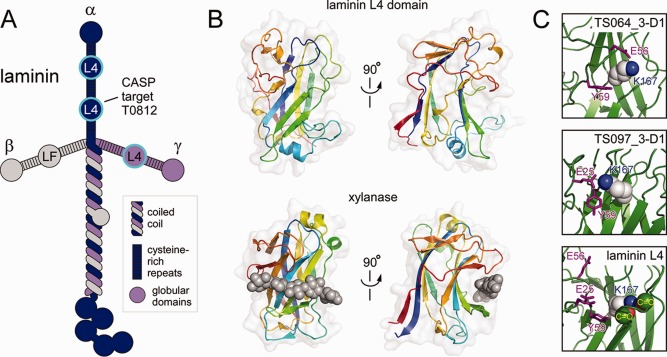
The laminin L4 domain. (**A**) Context of L4 domains within intact laminin. The position of the LF domain, another predicted CBM, is also shown. (**B**) (Top) Ribbon diagram (amino terminus red, carboxy terminus blue) of the high‐resolution structure of an L4 domain (PDB codes 4YEP and 4YEQ), viewed in two orientations. (Bottom) Structure of a carbohydrate‐binding protein in complex with oligosaccharide (PDB code 1GNY). The bound oligosaccharide is shown in gray space‐filling format. (**C**) Amino acid residue K167 (space‐filling format with the side‐chain nitrogen atom in blue) is in surface‐exposed positions interacting with acidic and aromatic amino acids (purple sticks) in CASP models (top and middle), whereas the K167 side‐chain is in fact buried in the laminin L4 structure (bottom) and found interacting with backbone carbonyl groups (yellow C=O labels).

The building blocks of many extracellular matrix (ECM) proteins are fiber‐forming coiled‐coil motifs and extended repeats of disulfide‐rich modules. Interspersed among these elongated structures are various globular domains, which contribute to the adhesive, network‐forming, or signaling activities of the ECM. In the ancient and widespread family of ECM proteins known as laminins, sets of tandem disulfide‐rich modules are interrupted at certain positions by globular domains of two types: LF domains and L4 domains [Fig. 2(A)].^9^ The purposes of these domains have not yet been revealed^9^ but the strong conservation of their presence and amino acid sequences throughout animal evolution suggests they make an important contribution to ECM function.

Prior to CASP11, a possible structural similarity between L4 domains and carbohydrate‐binding modules (CBMs) was proposed.^10^ Indeed, structural similarity between LF domains and CBMs is readily recognizable by threading (unpublished observations), but L4 domains show no obvious amino acid sequence homology with LF domains, and assignment of the L4 fold on the basis of amino acid sequence alone is not trivial. As validated now by X‐ray crystallography,^11^ L4 domains do belong to a β‐sandwich fold class shared with a superfamily of CBMs. As assessed using Dali,^12^ the laminin L4 domain gave a *Z* score of 9.7 and an RMSD for Cα atoms of 3.0 Å when compared with the closest match in the existing protein structure database, an endo‐1,4‐β‐xylanase with 153 amino acid residues (PDB code 1GNY) [Fig. 2(B)]. However, the laminin domain contains about 180 residues, whereas the Dali alignments span about 130 residues, so the templates offer only a partial solution to the modeling problem, and the actual L4 structure deviates substantially from other representatives of the fold. These factors placed the laminin L4 domain into the “hard” category of template‐based modeling in CASP. The high‐resolution crystal structure of a laminin L4 domain was required to reveal all the subtleties of its somewhat deviant β‐sandwich architecture [Fig. 2(B)].^11^

In the CASP11 experiment many of the models identified the correct β‐sandwich fold for the laminin L4 domain, but a large number also failed spectacularly, predicting elongated structures with two subdomains, or even all‐helical folds. The best model, submitted by the Baker group (TS064_3‐D1), reached a GDT_TS of 44 (all‐atom RMSD 6.5 Å). In this model, slightly more than 50% of the residues correctly align with the reference structure in a superposition generated with a 4 Å distance cutoff. Considering the submissions of all groups, most of the predictions partitioned clearly into those that identified the correct structure superfamily versus those that did not. A few predictions captured the correct fold but positioned the loops so wildly as to undermine the fold match. Another set of predictions identified a β‐sandwich fold but erred in the order of some of the β‐strands.

Notably, the difference between the top‐scoring models and those just slightly less accurate was the deviation from template structures. Specifically, the Baker model gave a *Z*‐score of only 5.8 and an RMSD for Cα atoms of 3.4 Å over 116 residues when compared with the template structure 1GNY. The Baker lab appears to have used template‐based modeling as a jumping‐off point rather than a restrictive end‐point. In contrast, another model, proposed by the RLuethy group (TS097_3‐D1), gave a *Z*‐score of 13.9 and a Cα RMSD of 2.6 Å over 164 aligned residues compared with a β‐agarase structure, demonstrating a tighter retention of the template structure at the expense of accurately modeling the novelty in the laminin L4 domain. The structural differences between the actual laminin L4 fold and other CBMs in the database were not sufficiently appreciated in many cases.

Some of the particular challenges offered by the L4 domain involve buried charged residues and exposed aromatic groups. For example, a lysine side‐chain (CASP residue K167; K1342 in the full laminin amino acid sequence) emerges from the outer face of one of the central β‐strands. The best CASP models placed this lysine in solvent‐exposed positions between glutamic acids and a tyrosine [Fig. 2(C)], the latter enabling a cation‐π interaction. In the crystal structure, however, the lysine side‐chain is buried by loops, interacting with backbone carbonyls [Fig. 2(C)]. Conversely, most modeling attempts succumbed to the reasonable temptation to bury hydrophobic side chains. However, the best model and the actual L4 structure point the phenylalanine and tyrosine side chains of a FXXY motif out toward solvent. In the crystallographic L4 structure, these aromatic side chains (F92 and Y95) line a surface cavity that may serve as a ligand binding site; no corresponding cavity exists in the predicted structures. A final source of error comprises the β‐sheet edge strands, which are positioned out of register even in the best model, such that inward‐ and outward‐facing residues are swapped. The lack of a clear alternating hydrophobic/polar pattern in the primary structure of these regions may be responsible for this break‐down.

In summary, the laminin L4 domain structure was an extremely demanding prediction task. The top model is, in many aspects, to be commended, but the devilish details have had their day.

### Snake adenovirus 1 fiber head (CASP: T0785; PDB: 4D0U, 4D1F, 4D1G, 4D0V, 4UMI)—provided by Abhimanyu K. Singh and Mark J. van Raaij

**Figure 3:**
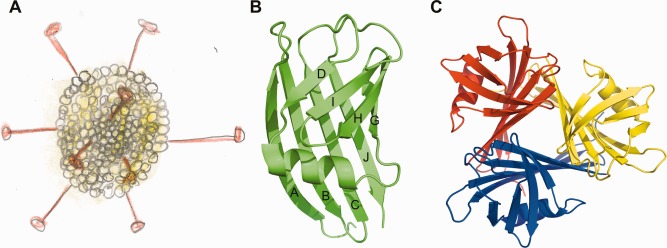
Snake Adenovirus 1 and its fiber head protein. (**A**) Schematic drawing of an icosahedral adenovirus with trimeric fiber proteins protruding from each of the twelve vertices. The head domains are located at the distal ends of the fibers. (**B** and **C**) Cartoon representation of a fiber head monomer (A) and a fiber head trimer (C). In part B the β‐strands are labeled. Parts B and C were prepared using the PyMOL Molecular Graphics System, Version 1.4.1, Schrödinger LLC and were first published in Singh 2014.^17^

Adenoviruses are important pathogens of vertebrates,^13^ but are also investigated to understand general mechanisms of molecular biology^14^ and used as vectors for gene and cancer therapy trials.^15^ Each of the twenty facets of the icosahedral adenovirus capsid is formed by twelve hexon protein trimers, while the twelve vertices are formed by the penton base proteins.^16^ Into each of the penton base pentamers, a trimeric fiber protein is inserted [Fig. 3(A)]; this fiber protein is responsible for the primary virus‐host interaction.^18^ Structurally, the fiber can be divided into three domains; an N‐terminal virus attachment or tail domain, a central shaft domain and a distal C‐terminal globular head or knob domain. The tail domain anchors the fiber to the penton base.^19^ The central shaft domain contains triple beta‐spiral sequence repeats, forming a thin, but stable, elongated structure.^20^, ^21^ Each monomer of the adenovirus fiber head trimer contains an eight‐stranded beta‐sandwich.^22^ The globular fiber head engages host receptors, while the shaft domain provides reach and flexibility.^23^

The family *Adenoviridae* has been subdivided into five genera:^24^ Mastadenovirus (infecting mammals, including humans), Aviadenovirus (infecting birds), Atadenovirus, Siadenovirus (infecting various hosts) and Ichtadenovirus (infecting fish). Adenoviruses from the Atadenovirus genus have been isolated from squamate reptile hosts, ruminants and birds and have a characteristic gene organization and capsid morphology. Snake Atadenovirus 1 was isolated from a corn snake (*Elaphe guttata*), which showed clinical signs of pneumonia.^25^ Snake Adenovirus 1 fiber has 345 amino acid residues;^26^ its carboxy‐terminal part has only between 12 and 18% sequence identity to adenovirus fiber heads of known structure. Potential beta‐spiral repeats 21 are present between residues 38 and 224. A putative loop region between residues 226 and 236 containing several prolines might separate the shaft from the head domain, leaving 111 residues for the head domain, shorter than all adenovirus fiber heads with known structures. The Ovine Atadenovirus D fiber head is also significantly smaller than other adenovirus fiber heads, as shown by electron microscopy.^27^ The crystal structure of the Snake Adenovirus 1 fiber head domain was determined by the multi‐wavelength anomalous dispersion method and refined at 1.33 Å resolution [Fig. 3(B,C)].^17^ This is the first Atadenovirus for which the structure of the fiber head has been determined. Despite the absence of significant sequence homology, the fiber head has the same beta‐sandwich propeller topology as other adenovirus fiber heads, with conservation of the ABCJ and GHID beta‐sheets (in Human Adenovirus 5 fiber head, the DG‐loop contains two additional beta‐strands E and F, but these are absent in the Snake Adenovirus 1 fiber head). However, the overall trimeric assembly is very compact, with a diameter of 4.6 nm and a height of 3.8 nm, compared with a diameter of 6.2 nm and a height of 4.0 nm for the Human Adenovirus 5.^22^ The AB‐, BC‐, GH‐, and HI connections are beta‐turns of two residues each. The CD‐ and IJ‐loops contain seven residues each, while the DG‐loop is composed of sixteen amino acid residues, containing an eight‐residue alpha‐helix.

Surprisingly, a structural homology search showed receptor binding proteins of bacteriophages P2 (PDB code 2BSE^28^) and TP901‐1 (PDB code 2F0C^29^) as the closest match. Further hits included the avian reovirus attachment protein sigma C (PDB code 2BT7^30^), the Human Adenovirus 37 and 19p fiber head domains (PDB codes 1UXA and 1UXB^31^), in descending order of similarity. The 99‐ and 98‐residue C‐terminal receptor binding domains of TP901‐1 and P2 bacteriophages are beta‐barrels made up of six anti‐parallel beta‐strands in case of the former and seven in case of the latter, with compact structures comparable in dimensions to the Snake Adenovirus 1 fiber head. The other known adenovirus fiber head structures all have longer loops. Besides loop length, the average number of residues per strand is also higher (10 vs. 8), which makes them taller.

The structure of the Snake Adenovirus 1 fiber head was difficult to predict due to the lack of significant sequence identity with any protein of known structure. In many cases the predicted structures contain significant amounts of alpha‐helices, while the target structure is mainly beta‐structured. The topologies of the predicted structures do not resemble the solved crystal structure, which means that predictions based on threading the new sequence on the chosen structural backbones, at least in this case, failed. It is possible that if a known adenovirus fiber head structure were to be used as a structural framework, predictions would have been more successful. It also appears the trimeric nature of the protein was not taken into account in the predictions, although this fact was provided as information with the target sequence.

If the conservation of topology (that is, the existence of ABCJ and GHID sheets) would have been foreseen, despite the lack of sequence homology, known adenovirus fiber head structures could have been used for more successful structure predictions. The smaller size of the fiber head might also have been foreseen from the electron microscopy experiments done on Ovine Atadenovirus D. If the fact that the protein forms a homo‐trimer would have been taken into account, predictions might also have been more accurate. Now that the structure of the first Atadenovirus fiber head domain is known, it should be possible to make reliable structure predictions for the homologous domains of other Atadenovirus fiber heads with high sequence homology, like the fiber 1 of Lizard Adenovirus 2, and perhaps also for Atadenovirus fiber heads with low sequence homology, like those of Bovine Adenovirus 4 and Ovine Adenovirus D. Apart from the fold, a major interest in determining the structure of the Snake Adenovirus 1 fiber head was to extract information about receptor‐binding. However, the receptor for Snake Adenovirus 1 is currently unknown and the structure did not reveal suggestive features, such as strongly negatively or positively charged regions.^17^ Therefore, further experiments are necessary to identify the receptor and determine its binding site.

### The structure of a novel biofilm‐dispersing nuclease NucB (CASP: T0824; PDB: N/A)—provided by Arnaud Baslé and Richard J. Lewis

Free‐living, motile bacteria can develop into a stationary, multicellular community of cells on natural or artificial moist surfaces; these communities are known as biofilms. Whereas biofilms are beneficial to bioremediation strategies, they are problematic in water and sewage treatment plants and pipes because they cause corrosion and clogging.^32^ Maintaining processing plant free of biofilms in the “white” biotech sector, which is dependent upon the intensive culturing of micro‐organisms, is a significant industrial challenge. Soil‐dwelling bacteria are associated with the biofilms of plants; whilst the nitrogen‐fixing *Rhizobium* exists symbiotically with the roots of plants, biofilms are involved in various diseases of fruit and vegetable crops.^33^ Medical implants and devices are frequently contaminated by biofilms, dental caries, and ear infections are caused by biofilms, and the persistence of chronic lung infections in cystic fibrosis patients is due to biofilms of *Pseudomonas*.^34^ Indeed, >65% of hospital‐acquired infections in the US are associated with biofilms, the annual treatment costs of which exceed $1 billion.^35^

The treatment of biofilms with antibiotics is not efficient as their penetration into biofilms is reduced by the extracellular matrix,^34^ an impermeable barrier comprising exopolysaccharide, amyloid‐like proteins and DNA that glues the biofilm together.^36^ Nearly 60 years ago Catlin demonstrated that the matrix contained DNA, and that the addition of bovine DNase‐I degraded the DNA in the extracellular matrix to result in biofilm dispersal.^36^ Subsequently, DNase‐I has been used to treat *Pseudomonas* biofilms in cystic fibrosis patients,^37^ but the effective treatment of biofilms in industrial, agricultural, societal, and healthcare settings requires rigorous addressing. The biofilm must be disrupted to return the bacteria to a free‐living, motile state, susceptible to the action of antibiotics. There are various genetic strategies employed by bacteria to regulate the synthesis of the biofilm,^38^ but a key element of biofilm dispersal is provided by a secreted DNase called NucB.^39^ NucB is a small protein of 109 amino acids, the sequence of which is dissimilar to all other structures in the PDB—the closest matches all have *E*‐values greater than 1. In order to understand how NucB functions to disperse biofilms, to gain insight into whether this enzyme acts either as an endo‐ or an exonuclease, and to determine the DNA sequence preference—if any—of NucB, its structure was solved by X‐ray crystallography with phases obtained by sulfur anomalous scattering.

**Figure 4:**
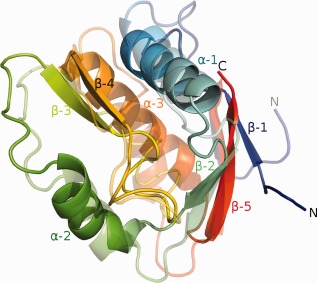
The NucB protein. A ribbon diagram of the crystal structure of NucB (solid colors) superimposed on the best prediction, TS064_2 (semitransparent colors). In both instances, the ribbon is color‐ramped from blue to red, corresponding to the N‐ and C‐termini, respectively.

The structure of NucB (Fig. 4) contains three α‐helices and five β‐strands in a single domain of two lobes; the smaller lobe comprises residues 36 to 80 (α‐helix 2, β‐strands 3 and 4) and the larger contains residues 2 to 35 (α‐helix 1 and β‐strands 1 and 2) and 84 to 109 (α‐helix 3 and β‐strand 5). The N‐ and C‐terminal residues are close in space and form a pair of β‐strands (1 and 5) that pack against each other in a parallel fashion against the anti‐parallel β‐strand 2. The NucB structure describes an approximate triangular pyramid with edge lengths of ∼25 Å; the base of the pyramid is formed by α‐helices 2 and 3, and the loop connecting α‐helix 2 to β‐strand 3, and the peak of the pyramid is formed by the C‐terminus of α‐helix 1. Inspection of the solvent‐accessible surface of NucB reveals that the flat base of the pyramid contains a 14 Å deep, 9 Å wide, 18 Å long depression that is formed mostly by conserved amino acids. This depression is necessary to accommodate a single strand of DNA and to present the scissile phosphodiester bond to the catalytic apparatus. The base of the depression is predominantly negatively‐charged, to interact with the bases of the DNA, whereas the lips of the cavity are mostly positively‐charged to interact with the phosphate backbone, and there is no molecular wall that one might imagine would be necessary to confer exo‐nuclease activity.

Perhaps unsurprisingly given the absence of structures with sequences similar to NucB in the PDB, structure‐based searches also failed to identify homologues of NucB with meaningful structural similarity. It is therefore impossible to answer any of the questions that presented themselves from the structure of NucB alone. That said, based upon the successful structural analysis of NucB, an aspartate in the pocket base was mutated, and the substitution of this amino acid with either asparagine or alanine resulted in a loss of nuclease activity. Therefore, the structure did enable the identification of the enzyme's active site and furthermore suggested that NucB is an endonuclease.

The best prediction on this target, model T0824TS064_2 from the Baker group, recapitulated many of the main features of NucB (Fig. 4) including the presence of five β‐strands and three α‐helices, their approximate location in the structure, the parallel packing of the first and last β‐strands (and the antiparallel packing of β‐strands 2 and 5) and the sole disulfide in the structure. This model appeared to be an exceptional prediction on such a challenging target scoring 55 GDT_TS points and outscoring models from the runner‐up Jones‐UCL group by 14 points and from all other groups by >22 GDT_TS points! The separation of the two best groups from the rest is most likely due to the successful application of new covariation contact prediction techniques that are being actively developed at the UC Washington and UC London groups.^6^, ^40^ It should be mentioned, however, that even in the best CASP model, α‐helices 2 and 3 and β‐strands 3 and 4 are displaced in comparison with the crystal structure such that the backbones vary by as much as 6.5 Å, especially in the vicinity of residues that our biochemical experiments have shown to be essential for the nuclease activity of the enzyme. Therefore, even though the best predictions in CASP11 were impressively close to the experimental structure, the critical functional details of this enzyme proved elusive to the predictors.

### A new protein domain associated with transmembrane solute transport and two component signal transduction (CASP: T0816; PDB: 5A1Q)—provided by Mateusz Korycinski, Marcus D. Hartmann, and Andrei N. Lupas

Sensory pathways frequently include transmembrane receptors as one of their components. These generally have a homodimeric architecture, consisting in its basic form of an N‐terminal extracellular sensor, transmembrane helices, and an intracellular effector. As an exception, an archaeal receptor family—exemplified by Af1503 from *Archaeoglobus fulgidus—*is C‐terminally shortened, lacking a recognizable effector module and having a HAMP domain as its sole cytosolic part. In studying Af1503‐like receptors we found that they are often genomically coupled to short proteins of about 60 to 90 residues—exemplified by Af1502. Af1502 itself has 68 residues and is encoded by the fourth gene in the *Af1505‐Af1502* operon, located on the minus strand of the *A. fulgidus* chromosome.^41^ Its gene is translationally coupled with the preceding gene encoding Af1503. The first gene in the operon, *Af1505*, encodes a putative metal‐ion transporter belonging to solute carrier family 41 (Mg^2+^‐transporter‐E, MgtE). Indeed, the genomic environment of Af1503‐like receptors is frequently enriched for components of membrane transport systems.

Sequence similarity searches using BLAST,^42^ HMMER,^43^ or HHblits^44^ fail to detect the similarity of Af1502 to the other proteins of its kind, due to its substantial divergence. Nevertheless, the homology of these proteins is supported by their genomic location, predicted secondary structure, patterns of hydrophobic residues, and a shared LGPx(x)A motif. Sequence profile searches further show that they are related to a domain found in a family of large, membrane‐associated proteins exemplified by the histidine kinase CbrA, a global regulator of metabolism, virulence, and antibiotic resistance in *Pseudomonas aeruginosa*.^45^, ^46^ Almost invariably, the domain connects membrane domains belonging to the sodium solute symporter family (SLC5) with cytosolic domains mediating two‐component signal transduction (TCST). We have therefore named it STAC (**S**LC5 and **T**CST‐**A**ssociated **C**omponent) and propose that it is involved in regulating solute transport.^47^ Given our long‐standing interest in Af1503 as a model system for transmembrane signal transduction, we have undertaken a biochemical and structural study of Af1502.

**Figure 5:**
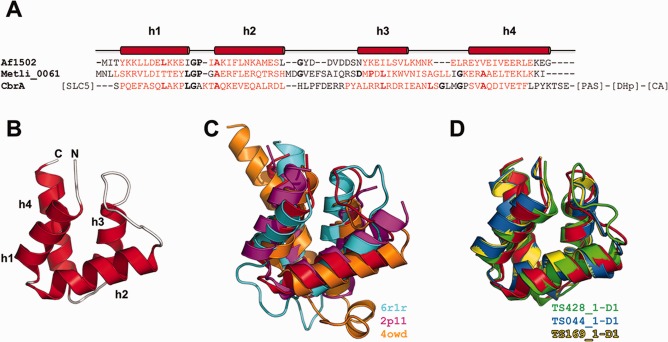
The Af1502 protein. (**A**) Sequence alignment of Af1502 with a stand‐alone STAC protein from *Methanofollis liminatans* and the STAC domain of the histidine kinase CbrA from *Pseudomonas aeruginosa*, colored according to the consensus secondary structure prediction (red = helix; black = loop). The observed secondary structure is shown above the alignment. Residues in bold characters are observed in a majority of STAC proteins. Further domains of CbrA are indicated in square brackets. (**B**) Crystal structure of Af1502 (PDB code 5A1Q). (**C**) Superimposition of Af1502 (red) to the best‐scoring DALI matches, as listed in the figure. All three matches are made to substructures within larger proteins. (**D**) Superimposition of Af1502 (red) to the best‐scoring predictions.

We predicted the secondary structure of Af1502 with the meta‐tool Quick2D in the MPI Bioinformatics Toolkit.^48^ The consensus prediction was of three helices, with the conserved LGPx(x)A motif connecting helices h1 and h2 [Fig. 5(A)]. However, the consensus prediction for the STAC domain family as a whole was of four helices. CD‐spectroscopy confirmed the α‐helical nature of the protein to a temperature of 95°C, showing that it is well folded and exquisitely stable. Structure determination of the SeMet‐derivative by X‐ray crystallography yielded a dataset to a resolution of 1.6 Å, showing a four‐helical bundle of two α‐hairpins, connected by a linker of nine residues [Fig. 5(B)]. The best‐diffracting crystals contained two monomers in the asymmetric unit, forming an extended interface of 580 Å^2^ via helices h2 and h3. Based on geometric criteria, the Evolutionary Protein‐Protein Interface Classifier (EPPIC)^49^ suggested that the observed interface might be biologically relevant. However, analyses performed by analytical gel filtration and static light scattering determined Af1502 as monomeric. Since STAC is either genetically coupled to dimeric receptors or an actual domain thereof, we explored this question further by NMR spectroscopy across a range of protein concentrations and observed some shift changes, however not at the potential dimer interface. We conclude that Af1502 is a monomer, with the crystallographic dimer caused by high protein concentration in the crystal.

A search for structurally similar domains using DALI^12^ yielded many matches with *Z‐*scores >2. This result is hardly surprising, considering the abundance of four‐helix bundles in proteins of known structure. The best matches were to the R1 subunit of ribonucleotide reductase (PDB: 6r1r, *Z*‐score: 6.0, Cα rmsd over 62 residues of 2.6 Å), a putative hydrolase of *Burkholderia xenovorans* (PDB: 2p11, *Z*‐score: 5.4, Cα rmsd over 60 residues of 2.7 Å) and the MltF protein of *Pseudomonas aeruginosa* (PDB: 3owd, *Z*‐score: 4.8, Cα rmsd over 61 residues of 2.8 Å) [Fig. 5(C)].

Despite its small size and the presence of many good templates in the structure database, Af1502 was not a trivial target, because it behaved as a singleton in sequence searches and its secondary structure prediction suggested a three‐helix bundle. Nonetheless, many accurate predictions were submitted, with 23 models obtaining GDT_TS scores above 70 (all but one from human predictors). The best‐scoring first models were proposed by the Laufer group (TS428_1‐D1, GDT_TS of 89.71, Cα rmsd over 68 residues of 1.54 Å), LEER group (TS044_1‐D1, GDT_TS of 74.63, Cα rmsd over 68 residues of 2.14 Å) and LEE group (TS169_1‐D1, GDT_TS of 73.90, Cα rmsd over 68 residues of 2.16 Å). These models are conspicuously better than the best structural matches in proteins of known structure [Fig. 5(D)]. Particularly the Laufer model reproduces very accurately all structural parameters, including the angles, distances and registers of the helical interactions; the only more pronounced departure is in the nine‐residue loop connecting the two hairpins (omitting these nine residues results in a Cα rmsd of 1.08 Å for the remaining chain). The three server‐generated first models above a GDT_TS score of 60 were by QUARK (TS499_1‐D1, GDT_TS of 64.71, Cα rmsd over 68 residues of 4.03 Å), FUSION (TS345_1‐D1, GDT_TS of 63.23, Cα rmsd over 68 residues of 4.19 Å), and MULTICOM‐NOVEL (TS041_1‐D1, GDT_TS of 62.13, Cα rmsd over 68 residues of 3.33 Å). All three are clearly worse than the best DALI matches.

### Monotreme lactation protein (MLP) (CASP: T0777; PDB: 4V00, 4V3J)—provided by Thomas S. Peat and Janet Newman

Monotremes (platypus and echidna) are extremely interesting creatures from an evolutionary standpoint and there was nothing which shared any sequence homology to this monotreme protein in the PDB. Monotremes lay eggs and, after a brief incubation period, hatchlings emerge and are nourished by milk secreted by nipple‐less mammary patches on the mother's abdomen. The milk is the sole nutrient and immune protection for the young until they are weaned. One of the novel components of monotreme milk (relative to mammalian milk) is the MLP protein. MLP is found in both platypus and echidna milk (and shares 94% identity between the species) and is highly expressed throughout the lactation period. MLP was found to be antibacterial against *Staphylococcus aureus* and commensal *Enterococcus faecalis*, but not against several other bacteria such as *E. coli* and *Pseudomonas aeruginosa*. It was predicted to an amphipathic, α‐helical protein, a common feature of antimicrobial proteins.

**Figure 6:**
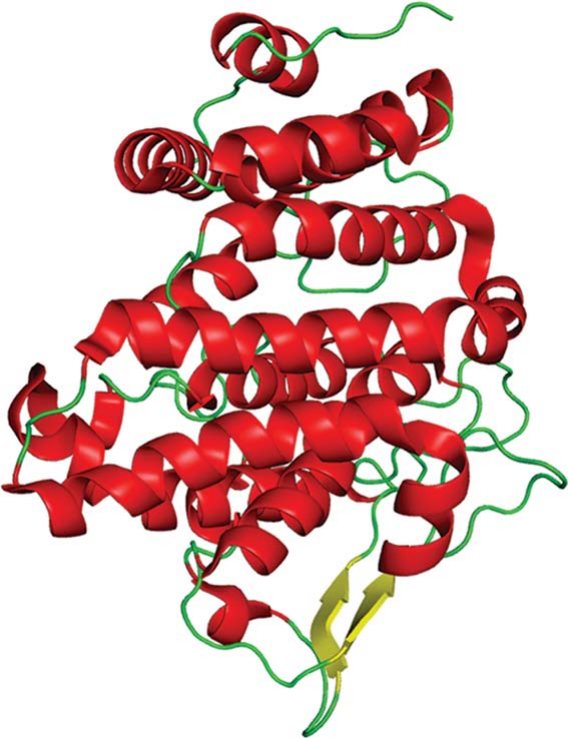
Cartoon representation of the monotreme lactation protein (MLP).

The protein was expressed (with a FLAG tag for purification) in cell culture (HEK293 cells) in order to retain potential post‐translational modifications and crystallized in three different space groups: P1, P2_1_, and C2. Both the P1 and C2 crystals diffracted beyond 2 Å and gave clear electron density maps that showed a single glycosylation site at Asn82. The P1 model is better ordered with all residues from 18 to 360 (or 362 for the second protomer in the asymmetric unit) with good backbone density except for a single loop between helices 11 and 12 (residues 197 to 203), which have higher B factors. The C2 model has several loops that are weak or missing in the structure. The structure is mostly α‐helical (13 helices) with just two short β‐strands (residues 50–54 and 156–160) in the N‐terminal half of the protein (Fig. 6). The protein structure has been compared with all other known structures in the Protein Data Bank using two different methods (PDBeFold and Dali) and no significant similarities were found.

Looking at just secondary structure predictions, the structure of MLP was predicted to be all α‐helical and except for the two short β‐strands, this is true. But being a novel sequence and a novel fold, there was little chance that the modelers would be able to predict the structure of this protein and this was borne out in the results: this protein appeared to be extremely difficult for prediction. All of the submitted models were of poor quality with GDT_TS scores of 17 or lower (that is, below the level of practical usability).

### Human vanin 1 protein (CASP: T0794; PDB: 4CYF, 4CYY)—provided by Thomas S. Peat and Janet Newman

Our interest in solving the structure of another CASP target—human vanin 1^50^—stemmed from its being a key enzyme linking metabolic disease and inflammation in the body. Vanin is involved in both coenzyme A catabolism (producing pantothenic acid (vitamin B5) and cysteamine from pantetheine) and inflammatory disease (for example, colitis). It is also an ectoenzyme (that is, found on the surface of the cell) and was originally discovered as a protein involved in leukocyte homing to the thymus. Bioinformatics suggested that the protein had two domains—a nitrilase enzymatic domain and a second, unknown domain. Nitrilases are generally found as dimers, so there was also a question of the quaternary structure of vanin 1.

**Figure 7:**
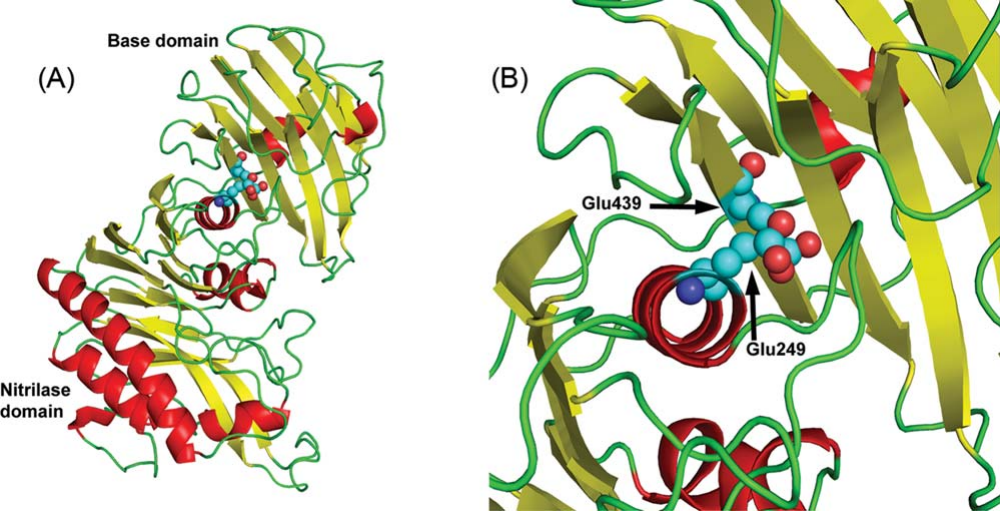
The vanin 1 protein. (**A**) The overall structure of human vanin 1 protein consisting of the N‐terminal nitrilase domain and the C‐terminal base domain. (**B**) One of the more interesting features of the structure—two glutamic residues, one from each domain, that are buried in the interface without any compensatory charges or other ions within hydrogen bond distance.

We produced the protein from cell culture (HEK293 cells) to give a protein with “native” post‐translational modifications (glycosylation) and activity. The wild‐type protein (minus the glycosylphosphatidyl inositol (GPI) anchor and with the addition of a FLAG tag for purification) was fully active in a specific assay and it crystallized in two different spacegroups (tetragonal P4_3_2_1_2 and trigonal P3_2_2). After solving the structure, the most unusual feature was the domain interface between the nitrilase enzymatic domain and what we refer to as the base domain (as it would be next to the cell surface anchored by the GPI). There are two buried glutamic acid residues, one from each domain—Glu249 and Glu439—that are within 4 Å of each other. The unusual aspect is that there are no compensating charges (Arg or Lys residues), no waters or metals and no obvious hydrogen bonding partners for these two residues (Fig. 7). It was hypothesized that these residues could have anomalous p*K*_a_'s and therefore be protonated (the crystallization conditions were at pH 6.3–6.5, two full pH units above the standard p*K*_a_ for glutamic acid). If these residues were protonated, it was reasoned that one (or both) could be mutated to glutamine and these mutations were made. The interesting outcome was that the mutant proteins (either Glu249Gln or Glu439Gln) were completely inactive despite the protein being well folded (shown through SAXS and DSF experiments).^50^ Clearly the activity of the protein depends on the relative orientation of these domains and this is dependent on the Glu249 and Glu439 residues being in close proximity during at least part of the enzymatic cycle.

The base domain has no sequence homology to any structure in the PDB, but it does have some structural homology to a lectin‐binding domain of a *Streptococcus pneumoniae* glycoside hydrolase (PDB code 2WMK), a protein involved in specific recognition of the Lewis antigen. This suggests that this base domain may be the functional domain that was described previously in leukocyte homing. It also suggests that the base domain can regulate the activity of the nitrilase domain through this domain interface and this may depend on what the base domain is bound to.

The basic fold of the nitrilase domain (N‐terminal domain) was predicted generally correctly (GDT_TS of 73 for the best models), although the sequence was often out of register due to differences in the length of the loops between the secondary structure elements. Loops of residues 37 to 48, 98 to 117, and 145 to 156 were modeled as being significantly shorter than the vanin crystal structure shows, and several of the models “made up” for these discrepancies by having a longer loop/extension around residues 183 to 184 of the N‐terminal vanin nitrilase domain. The C‐terminal domain (approximately residues 314–483) consists of almost entirely β‐strands, with two β‐sheets lying on top of each other with connecting loops (a curved β‐sandwich fold). Most of the models had a single β‐sheet with two long α‐helices (one at the C‐terminus) and various connecting loops. The fold as well as the orientation of the C‐terminal domain was basically incorrect (GDT_TS of models below 30). Another point of interest is that the C‐terminal (“base”) domain is tightly associated with the N‐terminal nitrilase domain and none of the models got this orientation/face correct. Potentially some of the models of the nitrilase domain could have given reasonable molecular replacement solutions, but none of the C‐terminal models could have been used to obtain a MR solution for the structure.

### An unknown phage protein, VCID6010, from the marine environment (CASP: T0820; PDB: N/A)—provided by Donald D. Lorimer, Timothy R. Craig, Victor Seguritan, Robert A. Edwards, Alex B. Burgin Jr, Forest Rohwer, and Anca M. Segall

It is estimated that there are more than 10^17^ viruses, including bacteriophages, in the world's oceans.^51^, ^52^ These viruses are poorly characterized and remain the largest reservoir of the Earth's unknown genetic diversity. Despite their simplicity and abundance, most phage sequences are too dissimilar from characterized proteins to allow for functional prediction. As a result, sequence similarity searching is insufficient for detecting viral structural proteins among the wealth of unknown viral sequences. By studying phage metagenomic sequences, we aim to uncover new enzymes with novel functions that could be exploited for various biotechnological purposes, including diagnostics as well as vaccine development.

**Figure 8:**
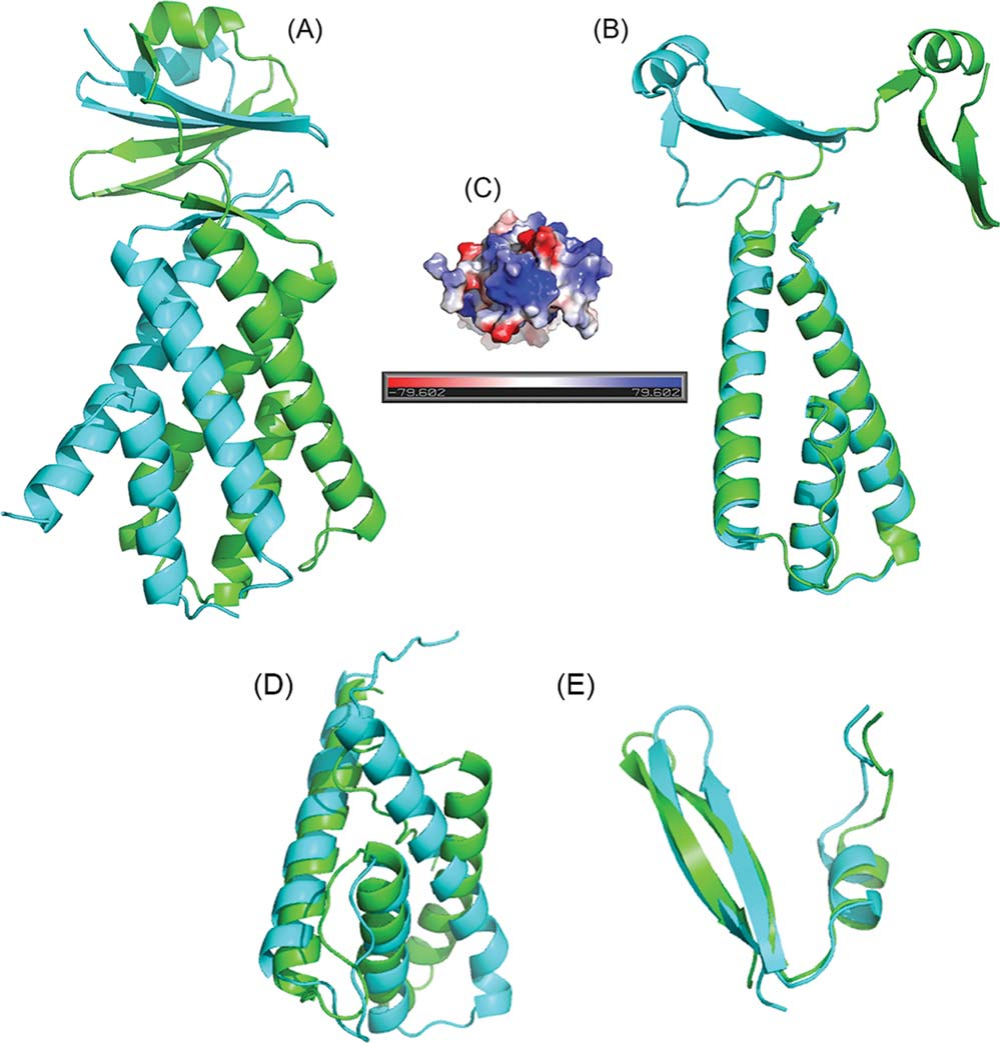
Cartoon representation of VCID6010. (**A**) This protein is composed of two domains: a lower helical domain and an upper β‐sheet containing domain. Each monomer in the dimer is colored differently to highlight the domain interactions and strand exchange. The lower, helical portion forms a teepee like shape composed of six helices. The upper domain of the protein contains a β‐sheet and is translated behind the axis of symmetry of the helical domain. (**B**) Overlay of chain A and chain B. The N‐terminal, α‐helical domains of the two chains overlay nearly perfectly whereas the C‐terminal are very dissimilar. Chain A is colored green and chain B is colored cyan. (**C**) Electrostatic charge distribution on VCID6010 showing a patch of positively charged residues on the bottom of the molecule. (**D**, **E**) CASP11 models (green) giving the best overlay with the VCID6010 structural domains (cyan). (D) Model T0820TS169_1‐D1 from the Lee group superimposed onto the N‐terminal domain; (E) model T0820TS328_1‐D2 from the RosEda group superimposed onto the C‐terminal domain. The figures were generated with PyMol (www.pymol.org).

This protein sequence was identified from a metagenomic pool of sequences isolated from the viral fraction of marine environmental samples. The metagenomic sequences were then analyzed with an artificial neural network to identify protein‐coding regions that serve structural roles in viruses.^53^ Based on the analysis, this particular sequence was predicted not to be a structural component of viruses. Highly pure protein was obtained for an expression construct, VCID6010. Crystallization trials were carried out at 16°C, and well‐diffracting crystals were obtained. A native dataset was collected to 2.35 Å. Unfortunately, the amino acid sequence of this protein has extremely low sequence identity to any previously solved structures currently deposited in the PDB (closest hit in PDB, 3F8T, *E*‐value = 0.87), which is one of the main reasons we believed that the structure would be an excellent candidate for CASP11. As no molecular replacement models were available, we attempted to generate a second dataset for SAD phasing using iodide ions.^54^ Unfortunately, the crystals did not survive the iodide soaking regime so SeMet‐labeled protein was prepared. A 2.05 Å dataset was collected and used to solve the structure. The SeMet model was used for molecular replacement with the 2.35 Å native data. The model shows a dimer with twofold symmetry with a shape we refer to as a teepee. Analytical HPLC confirms that this protein exists as a dimer in solution (data not shown). The lower portion of the each monomer is composed of three helices forming one half of the teepee, whereas in the upper portion each monomer is composed of one helix and four strands making an antiparallel β‐sheet with half of the strands donated by each monomer. As viewed face‐on [Fig. 8(A)] the lower part of the model looks symmetrical. The upper portion of the model is translated behind the axis of symmetry of the helical domain. Aligning chain A to chain B reveals the asymmetry of folding of the two chains [Fig. 8(B)]. The function of this protein in nature is unknown but we noticed that the bottom face has large patch of positively charged amino acids suggesting a possible role in binding to DNA or RNA [Fig. 8(C)]. Unfortunately, gel‐shift assays failed to demonstrate binding to single‐ or double‐stranded DNA or to tRNA (data not shown).

With low sequence identity to any known structures and non‐trivial composition of the dimer, this target as a whole appeared to be challenging for CASP participants. None of the models was able to correctly identify the dimeric nature of the structure or the extreme dissimilarity of folding of the two chains within the model. At the domain level, the best models for the first domain (residues 1–91) earned GDT_TS score of around 50, indicating that approximately half of the domain (helices 2 and 3) was modeled with an acceptable quality, while the rest (helix 1 and the loops) was modeled poorly, resulting in an overall high Cα rmsd of 7.3 Å [Fig. 8(D)]. The second domain (an alpha helix followed by two antiparallel β‐strands) is much shorter (36 residues), has homologues in the structural databases (for example, 3s31A), and thus was modeled substantially better with the best models reaching GDT_TS score of 83 [Fig. 8(E)].

### PilA1, the major Type IV pilin of *Clostridium difficile* NAP08 (CASP: T0803, PDB: 4OGM)—provided by Kurt Piepenbrink and Eric J. Sundberg

Type IV pili are a class of fibrous extracellular appendages found in both Gram‐negative and Gram‐positive bacteria, as well as archaea.^55^ All functions of Type IV pili are driven by adhesion of one kind or another and include horizontal gene transfer, host‐cell adhesion, and microcolony/biofilm formation. They are formed by helical assembly of protein subunits called pilins, driven by noncovalent interactions, particularly hydrophobic interactions between the subunit N‐termini. Each type IV pilin contains a signal peptide that is processed by a peptidase called PilD followed by a hydrophobic N‐terminal α‐helix (α1‐N), similar to a transmembrane domain, and a globular head‐domain. The head domains universally contain an α‐helical backbone (α1‐C) and a central antiparallel β‐sheet with at least four strands. All type IV pilins from Gram‐negative bacteria contain a disulfide bond, typically toward the C‐terminus, which is thought to stabilize the fold. Gram‐negative Type IV pili have also been subdivided into two classes, Type IVa and Type IVb, based on a variety of factors, including size, the length of the signal peptide and the identity of the first residue after the signal peptide (typically phenylalanine for Type IVa and a different aliphatic residue for Type IVb).^56^ Type IVa pili are found in a wide variety of organisms while Type IVb pili have been found primarily in enteric pathogenic bacteria such as enteropathogenic, enterohemorrhagic, and enterotoxigenic *Escherichia coli* and *Vibrio cholera*.^57^, ^58^

The type IV pili of Gram‐positive bacteria, including *Clostridium difficile*, are substantially less well characterized. However, in the past 5 years, several Gram‐positive bacteria have been demonstrated to produce Type IV pili^59^, ^60^, ^61^ and with the advent of widespread whole‐genome sequencing, genes for Type IV pilins and pilus biogenesis proteins have also been discovered in every member of the genus *Clostridia*. *C. difficile* produces Type IV pili consisting primarily of PilA1 but also incorporating at least one minor pilin, PilJ.^62^ The genome of *C. difficile* includes genes for a total of nine putative Type IV pilins, the majority of which are in three distinct gene clusters.^63^ The sequences of these pilins contain several unusual features; in the case of PilA1, there are no cysteine residues, indicating that it uses some other mechanism for stabilization. In 2014, the X‐ray crystal structure of PilJ, a minor pilin from *C. difficile* became the first high‐resolution structure of a pilin from a Gram‐positive bacterium^62^ and the structure of PilA1 is now the first of a major pilin from a Gram‐positive organism.^64^

**Figure 9:**
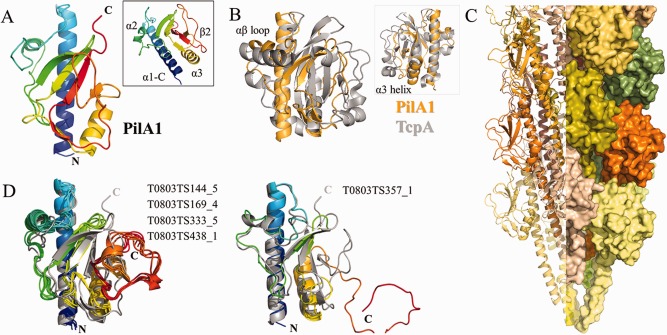
PilA1 from *Clostridium difficile*. (**A**) Ribbon diagram of PilA1 colored in a gradient from blue (N‐terminus) to red (C‐terminus). The inset panel shows the novel β2 sheet. (**B**) Superposition of PilA1 (gold) and TcpA (gray). The inset panel shows the reverse side, highlighting the similarity of the position of the α3 helix in the two pilins. (**C**) Model of a pilus fiber composed of PilA1; each subunit is colored individually. (**D**) CASP models, colored in gradients from blue (N‐terminus) to red (C‐terminus) superimposed onto the PilA1 crystal structure (gray). The figure was created in PyMol.

The overall fold of the soluble pilin head‐group of PilA1 follows the pattern seen in the Type IV pilins from Gram‐negative bacteria. The initial α‐helix and the central β‐sheet are clearly recognizable [Fig. 9(A)]. The loop between the α1‐C helix and the first strand of the central β‐sheet (αβ loop) contains a short α‐helix (α2) which is typical of Type IVb pilins but is also found in many Type IVa pilins.^55^ The central β‐sheet contains four anti‐parallel strands but, importantly, is discontinuous; that is, the order of the strands from one end of the sheet to the other is different from the order in which they occur in the protein sequence. This discontinuity is a hallmark of Type IVb pilins and may also help to explain some of the difficulties encountered in predicting the structure of PilA1 (see below). The most novel structural feature of PilA1 is the inclusion of a two‐stranded antiparallel β‐sheet below the central β‐sheet that we term the β2 sheet. The inclusion of additional β‐sheets is not unprecedented in Type IV pilins; notably the major pilin of PAK *Pseudomonas aeruginosa* contains a two‐stranded sheet in its αβ‐loop.^65^ However the position of the PilA1 β2 sheet is unique and may offer an explanation for how the fold of PilA1 is stabilized in the absence of disulfide bonds. PilA1 also contains a C‐terminal α‐helix (α3) in a position similar to that of many Type IVb pilins, including TcpA of *Vibrio cholerae* [Fig. 9(B)].^66^ Taken together, the structural similarities between PilA1 and the Type IVb pilins suggest that there is a functional similarity between the Type IV pili of *C. difficile* and those of other enteric pathogens including *Vibrio cholerae*, *Salmonella typhi* and enterohemorrhagic *E. coli* (EHEC). A model of the assembled pilus fiber is depicted in Figure 9(C).

For the participants of CASP11, predicting the structure of PilA1 proved to be a matter of halves; they were all much more successful on the N‐terminal half of the protein than the C‐terminal half. A structural alignment of the best scoring model with the X‐ray crystal structure identified 47 C_α_ pairs within 1 Å; 43 of these were in the N‐terminal portion of the structure (prior to the first strand of the β2 sheet). Despite the overall conservation of the Type IV pilin fold, the sequence identity between PilA1 and potential template structures in the PDB is in the range of 10 to 20% (the sequence identity between PilA1 and TcpA is ∼13%). This has obvious implications for structure prediction, particularly as the sequence similarity is highest at the N‐terminus and lessens steadily toward the C‐terminus. Perhaps as a consequence, of the five top models submitted to CASP11, all correctly predict the overall fold of PilA1 from the N‐terminus through the first two strands of the β‐sheet. All but T0803TS357_1 are nearly identical [Fig. 9(D)] and overestimate the helical character of the αβ loop, possibly because the previously solved Type IVb pilins were used as templates [Fig. 9(B)]. None of the top five models was able to model the C‐terminal portion of the protein successfully; the latter two strands of the central β‐sheet are not present and the C‐terminus is generally not tightly packed, particularly in T0803TS357_1, where it is extended to the point of being unfolded. However, all five models include the α3 helix in approximately the correct position, aided perhaps by its conservation in previously‐solved Type IVb pilin structures [Fig. 9(B)]. In all cases, the β2 sheet is not assembled, which prevents the formation of the remainder of the central β‐sheet. The absence of the β2 sheet in all five of the top predictions is not surprising given its novelty, but it may indicate a significant gap in our ability to translate predictions of secondary structure into predictions of tertiary structure. PSIPRED predicts the alpha and beta regions of PilA1 nearly perfectly but predicting the interactions that fold those regions into the tertiary structure has proven to be considerably more difficult.

### Final remarks

With the shift in CASP assessment to a more function‐oriented analysis, we hope that this manuscript will help future CASP assessors to identify relevant biological questions and guide them in selection of appropriate evaluation approaches. We hope that method developers will better understand which features of structures are important from the point of view of crystallographers and NMR spectroscopists, and how these features should be taken into account to develop better prediction tools. We also hope that structure providers will become better informed about abilities and limitations of new improved techniques in the field of protein structure prediction and use these techniques to their advantage. Finally, we hope that articles of this nature will pave the road for a more close symbiosis between all branches of the CASP process. Using the word “symbiosis” we wanted to emphasize that relations between the experimental structural biology and computational biology communities can be *mutually* beneficial. Not only are targets from the experimental community needed for development and testing of structure prediction methods, but also results of these methods can be practically helpful for experimental structure determination. As an example, we want to mention CASP11 target T0839 (the SLA2 adaptor protein involved in endocytosis), which was solved with molecular replacement using CASP‐submitted models (Rob Meijers, EMBL Hamburg outstation, article in preparation). In general, it has been shown in CASP^67^ and elsewhere^68^ that modeling can be effective in X‐ray crystallography by providing structures for molecular replacement, and in NMR spectroscopy for the development of high‐throughput structure determination methods.^69^
